# Growth Parameter Components of Adaptive Specificity during Experimental Evolution of the UVR-Inducible Mutator *Pseudomonas cichorii* 302959

**DOI:** 10.1371/journal.pone.0015975

**Published:** 2011-01-14

**Authors:** Michael R. Weigand, Vinh N. Tran, George W. Sundin

**Affiliations:** 1 Program in Genetics, Michigan State University, East Lansing, Michigan, United States of America; 2 Department of Plant Pathology, Michigan State University, East Lansing, Michigan, United States of America; 3 Centers for Microbial Ecology and Pathogenesis, Michigan State University, East Lansing, Michigan, United States of America; Laboratory of Human Bacterial Pathogenesis, National Institutes of Health, United States of America

## Abstract

**Background:**

Mutagenic DNA repair (MDR) transiently increases mutation rate through the activation of low-fidelity repair polymerases in response to specific, DNA-damaging environmental stress conditions such as ultraviolet radiation (UVR) exposure. These repair polymerases also confer UVR tolerance, intimately linking mutability and survival in bacteria that colone habitats subject to regular UVR exposure.

**Methodology/Principal Findings:**

Here, we investigate adaptive specificity in experimental lineages of the highly UVR-mutable epiphytic plant pathogen *Pseudomonas cichorii* 302959. Relative fitness measurements of isolates and population samples from replicate lineages indicated that adaptive improvements emerged early in all lineages of our evolution experiment and specific increases in relative fitness correlated with distinct improvements in doubling and lag times. Adaptive improvements gained under UVR and non-UVR conditions were acquired preferentially, and differentially contributed to relative fitness under varied growth conditions.

**Conclusions:**

These results support our earlier observations that MDR activation may contribute to gains in relative fitness without impeding normal patterns of adaptive specificity in *P. cichorii* 302959.

## Introduction

Mutation is the most fundamental source of variation on which natural selection may drive evolution. However, the predominantly deleterious nature of mutation maintains selection pressure in favor of lower mutation rates as evidenced by the conservation of multiple DNA error-avoidance and error-repair processes [1]. Yet hypermutator (or mutator) strains of bacteria have been observed in a variety of clinical, environmental, and laboratory populations with mutation rates 100-1000 times greater than wild-type due to defects in DNA proofreading and repair machinery [2]. The widespread existence of both constitutive and inducible mutator genotypes suggests that evolutionary strategies of bacteria include mechanisms for increasing mutability [3], [4]. These mutator genotypes have not been shown to confer an intrinsic fitness advantage and their abundance within natural populations likely results from hitchhiking with secondary, beneficial mutations which counterbalance the cost of accumulating deleterious mutations [5], [6].

Inducible mutability in the form of mutagenic DNA repair (MDR) transiently increases mutation rate through the activation of low-fidelity repair polymerases in response to specific environmental stress conditions such as ultraviolet radiation (UVR) exposure. UVR directly damages DNA causing lesions, including cyclobutane pyrimidine dimers and 6-4 photoproducts, that distort the helical structure, interrupting replication fork progression and inducing the SOS response. The SOS regulon coordinates the control of more than 40 unlinked genes involved in DNA repair, recombination, and cell cycle control [7], [8]. Among these, the Y family DNA polymerases polIV (*Escherichia coli* DinB) and polV (*E. coli* UmuDC) rescue stalled replication forks and permit DNA synthesis across damaged regions of DNA in a template-independent manner by nature of their high processivity and low fidelity [9]. Secondary mutations caused by these error-prone polymerases comprise the majority of sequence alterations derived from UVR exposure and define the inducible mutator phenotype. This translesion DNA synthesis activity confers both UVR tolerance and inducible mutability, intimately linking survival and mutation in habitats subject to regular UVR exposure.

MDR-mediated UVR tolerance provides a critical ecological advantage to epiphytic plant pathogens harboring Y family polymerases that reside in habitats optimized for solar UVR exposure [10], [11]. We have previously reported our studies of experimental evolution with the highly UVR-mutable celery pathogen *Pseudomonas cichorii* 302959 [12]. The major contributor of UVR-induced mutability in this strain is *rulAB*, a homolog of *umuDC* found in various plant-associated *Pseudomonas* species [13]. This system is both ecologically relevant and experimentally tractable, making it ideally suited to investigate the influence of MDR on adaptation. In our previous study, parallel lineages of initially isogenic *P. cichorii* 302959 were maintained in a serial transfer regime for 500 generations during which half received daily MDR activation in the form of UVR exposure [12]. During that evolution experiment, mutations that conferred any competitive advantage under the lineage conditions were favored by natural selection.

The results of our initial study suggested that regular activation of inducible mutability in the form of MDR was not detrimental to fitness, but rather could contribute to adaptation and genetic diversity [12]. Another recent study has also suggested that when fitness improvements are available, adaptation may be facilitated by moderately enhanced mutagenesis [14]. In our previous study, relative fitness measurements of UVR-exposed lineages indicated a specificity of adaptation observable as higher fitness gains under UVR conditions than under non-UVR conditions that could not be attributed to increases in UVR tolerance alone. Conversely, lineages not exposed to daily UVR displayed comparable improvements in relative fitness under both UVR and non-UVR conditions. Such specialization is a general feature of adaptive evolution [15], [16]. However, given the common growth medium in both the UVR and non-UVR lineage environments, we expected that beneficial mutations would contribute to enhanced growth under both conditions.

In the present study, we investigated adaptive specificity in experimental lineages of *P. cichorii* 302959 by examining physiological components of relative fitness improvements. Relative fitness measurements indicate an overall growth advantage that we hypothesized could be subdivided into discrete improvements in bacterial physiology to reveal a more detailed understanding of the adaptation of *P. cichorii* 302959 to specific laboratory environments. Our results suggest that improvements in doubling and lag times contributed to specific relative fitness gains by both UVR and non-UVR lineages in their respective environments. Surprisingly, specific fitness improvements appeared relatively early in all experimental lineages, independent of regular MDR activation.

## Materials and Methods

### Bacterial strains, growth conditions, and general molecular biology techniques

The ancestral ‘round’ strain of *P. cichorii* 302959 was originally isolated in Japan [13]. In a previous study, sixteen populations of *P. cichorii* 302959 were derived from a single ancestral colony and propagated by serial transfer for 500 generations in minimal liquid media [12]. Eight UVR lineages (numbered 25–32) received a single, daily dose of UVC (254 nm) radiation to activate mutagenic DNA repair. Cultures were individually mixed 1∶1 with saline (0.85% NaCl) in a glass Petri dish and exposed to ∼40 J m^−2^ of UVC (254 nm) radiation from an XX-15 UV lamp (UVP Products, San Gabriel, CA) that resulted in ∼10% survival by each lineage population. The energy output of the lamp was monitored with a UV-X radiometer fitted with a UV-25 sensor (UVP Products, San Gabriel, CA) and determined to be 1.5 J m^−2^ s^−1^. Following UVR irradiation, cultures were diluted 1∶100 into fresh medium and incubated under dark conditions to minimize photoreactivation. These lineages diversified into a fluctuating coexistence of ‘round’ (R) and ‘fuzzy’ (F) colony morphotypes. Eight non-UVR lineages (numbered 33–40) were diluted daily into fresh medium without MDR activation by UVR exposure and contained only the R morphotype. The culture transfer strategies for both UVR and non-UVR lineages resulted in a 1000-fold daily increase in the growth of each population, representing ∼10 generations of binary fission.

**Figure 1:**
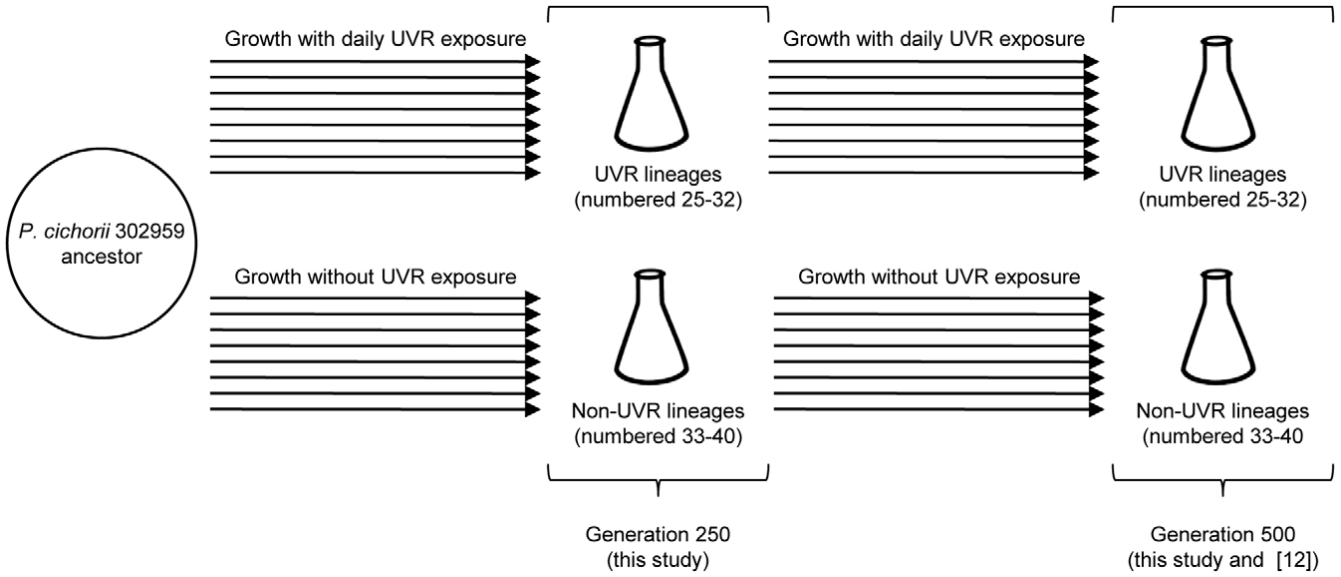
Evolutionary history of isolates and population samples derived from experimental lineages of *P. cichorii* 302959 and characterized in this study. Sixteen parallel lineages of *P. cichorii* 302959 were derived from a single colony and propagated by serial transfer. Eight UVR lineages received daily UVR exposure and diversified into mixtures of ‘round’ and ‘fuzzy’ colony morphotypes. Eight non-UVR lineages did not receive UVR exposure and contained only the ancestral, ‘round’ colony morphotype. All 16 lineages were propagated for 500 generations and samples from each were periodically preserved. Isolates and population samples in this study were derived from the ancestor following either 250 or 500 generations of selection in the evolution experiment.

Samples from each lineage were periodically preserved in a nonevolving state in 10% glycerol at −80°C. All evolved isolates in this study were derived from the ancestral genotype following either 250 or 500 generations of selection in the lineage experiment (Figure 1). We define “lineage isolates” as single-colony isolates from any of the replicate lineages in this experiment. Evolved isolates are denoted by lineage replicate and morphotype (*e.g.*, 25R refers to an isolate from lineage 25 that exhibits the round colony morphotype). Each lineage contained ∼10^10^ total *P. cichorii* 302959 cells at stationary phase, and the relative fitness of these communities has been shown to rely on the ecological interactions of sympatric genotypes [12]. Therefore, lineage communities were also analyzed in this study as population samples; these samples contained ∼10^8^ cells thawed from preserved UVR lineages to maintain the assemblage of diverse genotypes. Isolates and population samples from generation 500 characterized here are identical to those reported previously [12].

Strains of *P. cichorii* were cultured at 28°C in Luria–Burtani (LB) broth (Difco, Detroit, MI), in Davis Minimal broth supplemented with 25 mg l^−1^ glucose (DM25) (Difco), or on King's medium B (KB) agar [17]. Antibiotics were used where appropriate at the following concentrations: carbenicillin 50 µg ml^−1^, gentamicin 10 µg ml^−1^. Competent cell preparation was performed according to Choi *et al.* [18]. Plasmid DNA was isolated using the QIAprep Miniprep kit (QIAGEN, Valencia, CA). Transformation by electroporation, standard agarose gel electrophoresis, and other recombinant DNA techniques were performed according to Sambrook *et al.* [19].

### Competition experiments and relative fitness calculations

The relative fitness of all isolates and population samples was determined by direct competition with the ancestor under both UVR and non-UVR conditions to identify patterns of specific or general adaptation as described previously [12]. Briefly, competitions were performed under the same UVR and non-UVR conditions as the lineage experiment described above. To ensure that competitors were comparably acclimated to the competition environment, isolates were simultaneously removed from glycerol stocks maintained at −80°C, individually grown in LB broth overnight, and then individually grown for 24 hr in the competition environment. Competitors were mixed at a 1∶1 volumetric ratio, and appropriate dilutions were spread on KB agar in triplicate at 0 and 24 hr to estimate the initial and final density of each. Strain differentiation was accomplished by plasmid-encoded catechol 2,3-dioxygenase (*xylE*) which causes expressing colonies on agar media to turn yellow when sprayed with 0.1 M catechol. The relative fitness (*W*) of the two competitors was calculated as the ratio of their realized growth rate as described previously [12]. When the two competitors are equally fit in the competition environment, *W* = 1.

The six replicates of each relative fitness measurement were analyzed by two-tailed, independent *t*-tests against the hypothesis of mean equal fitness (*W* = 1). Fitness measurements were compared using two-tailed paired *t*-tests to determine any significant differences in adaptation between growth intervals (generations 250 vs. 500) or growth environments (UVR vs. non-UVR conditions).

### Growth curves and calculations

Before optical density (OD) measurement, lineage isolates and population samples were grown overnight in LB broth and subcultured into 10 ml DM25 at 28°C for 24 hr acclimation. Cultures were diluted 1000-fold into fresh DM25, and 100 µl samples were removed for OD measurement at 600-nm in 96-well microtiter plates using a Tecan Safire (Tecan US, Inc., Durham, NC). Samples were removed for measurement at 0 hr and at 1 hr intervals from 4 hr to 15 hr. Growth curves for each lineage isolate and population sample were repeated in triplicate under both UVR and non-UVR conditions.

OD_600_ values from growth curves were standardized by dividing by the initial OD_600_ (0 hr) and then log_2_ transformed. The transformed values were plotted, and the window of exponential growth was identified by linear regression [20]. Lag time was calculated simply as the x-intercept of the linear equation. Doubling time (g) was determined by the equation:


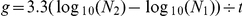


where *N*_1_ and *N*_2_ are raw, untransformed OD values corresponding to the window of exponential growth observed in the log_2_ transformation plot and *t* is the time interval between them [21].

### UVR tolerance assays

Lineage isolates and population samples were grown overnight in LB broth and 2 ml of culture were pelleted, washed with 1 ml saline, resuspended in 1 ml saline, and held on ice. The cell suspensions were mixed with 9 ml saline in a glass petri dish and exposed to a single dose of approximately 140 J m^−2^ UVC as described above. Cell suspensions were mixed continuously while receiving UVR doses to eliminate survival as a result of shading. Following irradiation, surviving cells were enumerated by dilution plating conducted under dark conditions.

## Results

### Relative fitness of evolved lineages

The relative fitness of all lineages was determined by competition with the *P. cichorii* 302959 ancestor under both UVR and non-UVR conditions to examine adaptation during experimental evolution. We evaluated the relative fitness of each lineage by competing both population samples (a subset of the entire lineage maintaining the assemblage of diverse genotypes) and single-colony isolates. In our previous study, we analyzed changes in relative fitness in replicate lineages after 500 generations [12]. In this current study, we report on alterations in relative fitness occurring after 250 generations with a focus on underlying mechanistic changes responsible for enhancement of relative fitness.

**Figure 2:**
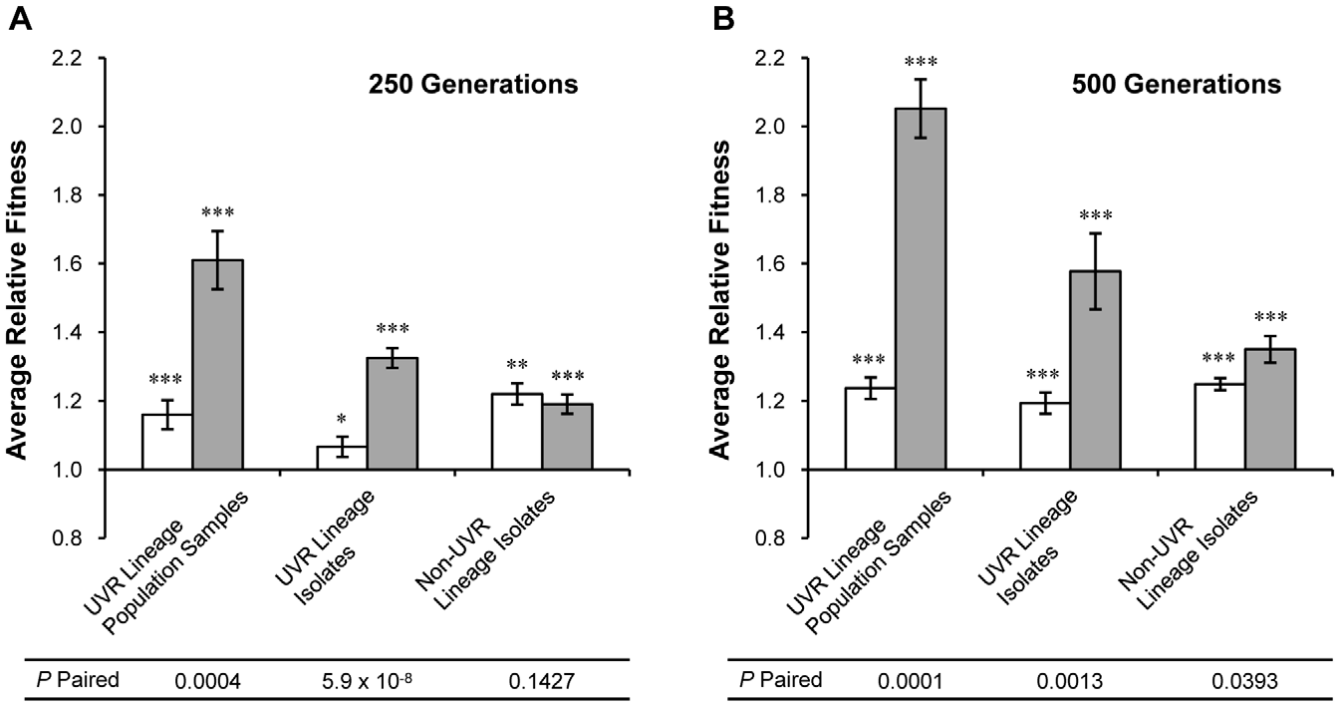
Average relative fitness indicates adaptive specificity in evolved lineages of *P. cichorii* 302959. Relative fitness of UVR lineage population samples, UVR lineage isolates, and non-UVR lineage isolates was measured by direct competition with the ancestor under non-UVR (open bars) and UVR (shaded bars) conditions after (A) 250 and (B) 500 generations of experimental evolution. Fitness values are means and error bars represent standard error of the mean. n = 8 for UVR lineage population samples and non-UVR lineage isolates but n = 16 for UVR lineage isolates. Values are significant by two-tailed independent *t*-test (α = 0.05) where indicated (* *P*<0.05, ** *P*<0.01, *** *P*<0.001). *P*-paired values correspond to two-tailed paired *t*-tests (α = 0.05) between average relative fitness values under the two conditions.

**Figure 3:**
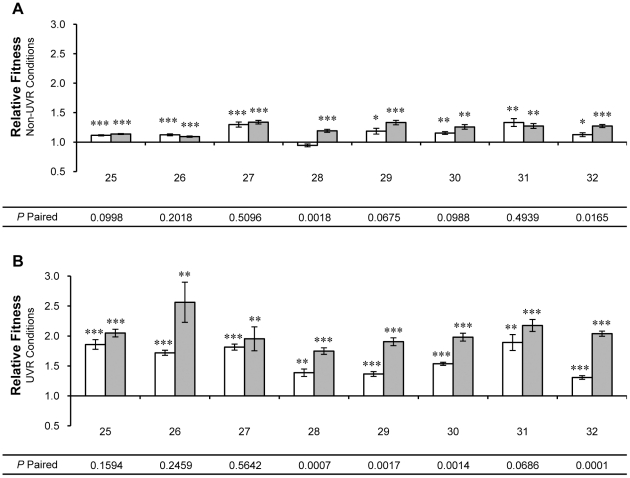
Relative fitness of individual population samples from UVR lineages 25-32. Relative fitness was measured by direct competition with the ancestor under (A) non-UVR and (B) UVR conditions. Population samples contained ∼10^8^ cells of *P. cichorii* 302959 after 250 (open bars) and 500 (shaded bars) generations of experimental evolution. Fitness values are means and error bars represent standard error of the mean. Values are significant by two-tailed independent *t*-test (d.f. = 5, α = 0.05) where indicated (* *P*<0.05, ** *P*<0.01, *** *P*<0.001). *P*-paired values correspond to two-tailed paired *t*-tests (d.f. = 5, α = 0.05) between relative fitness values at generation 250 and 500.

**Figure 4:**
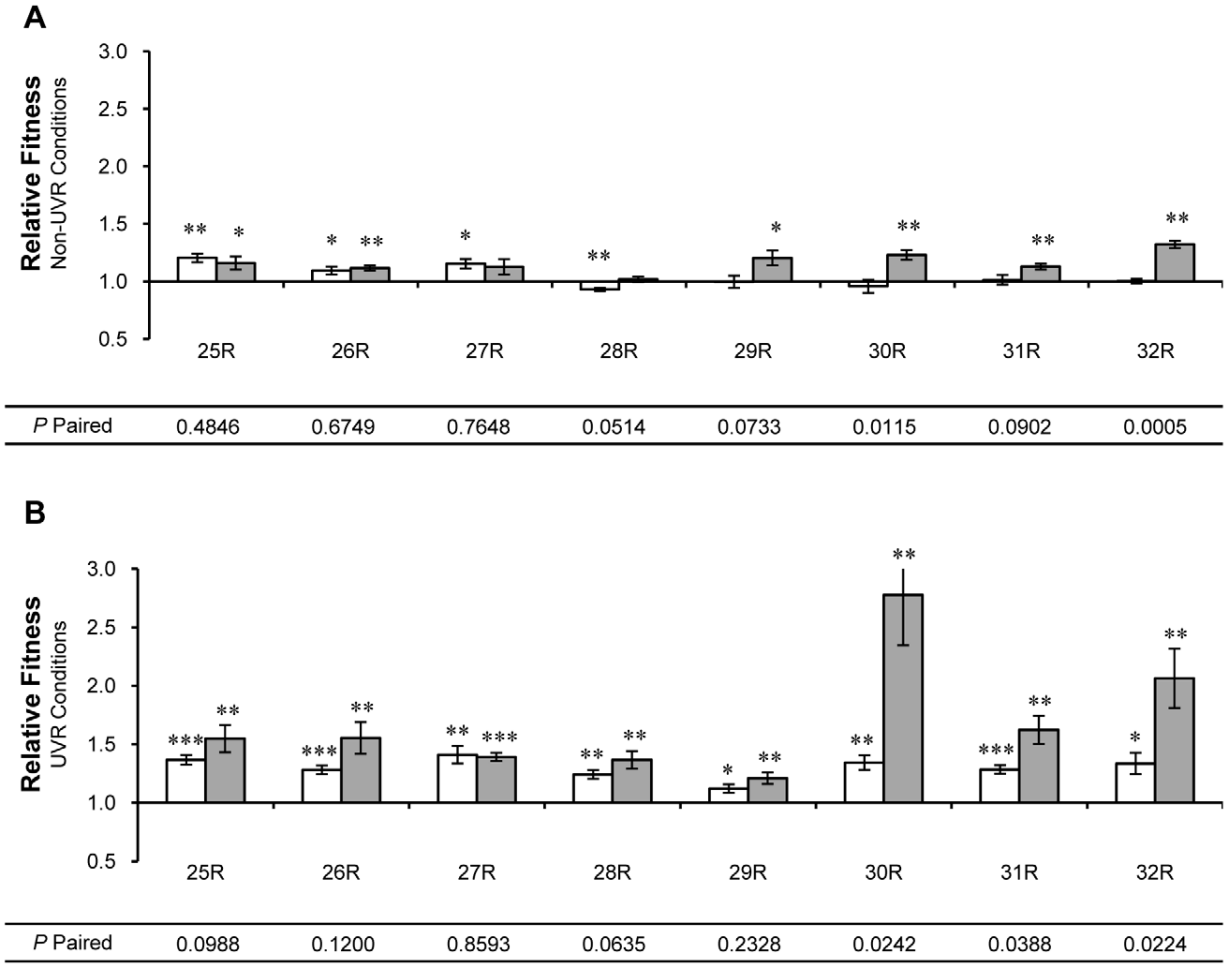
Relative fitness of ‘round’ (R) isolates from UVR lineages 25-32. Relative fitness was measured by direct competition with the ancestor under (A) non-UVR and (B) UVR conditions. Single isolates of *P. cichorii* 302959 exhibiting the ‘round’ colony morphology were taken after 250 (open bars) and 500 (shaded bars) generations of experimental evolution. Fitness values are means and error bars represent standard error of the mean. Values are significant by two-tailed independent *t*-test (d.f. = 5, α = 0.05) where indicated (* *P*<0.05, ** *P*<0.01, *** *P*<0.001). *P*-paired values correspond to two-tailed paired *t*-tests (d.f. = 5, α = 0.05) between relative fitness values for isolates from generation 250 and 500.

**Figure 5:**
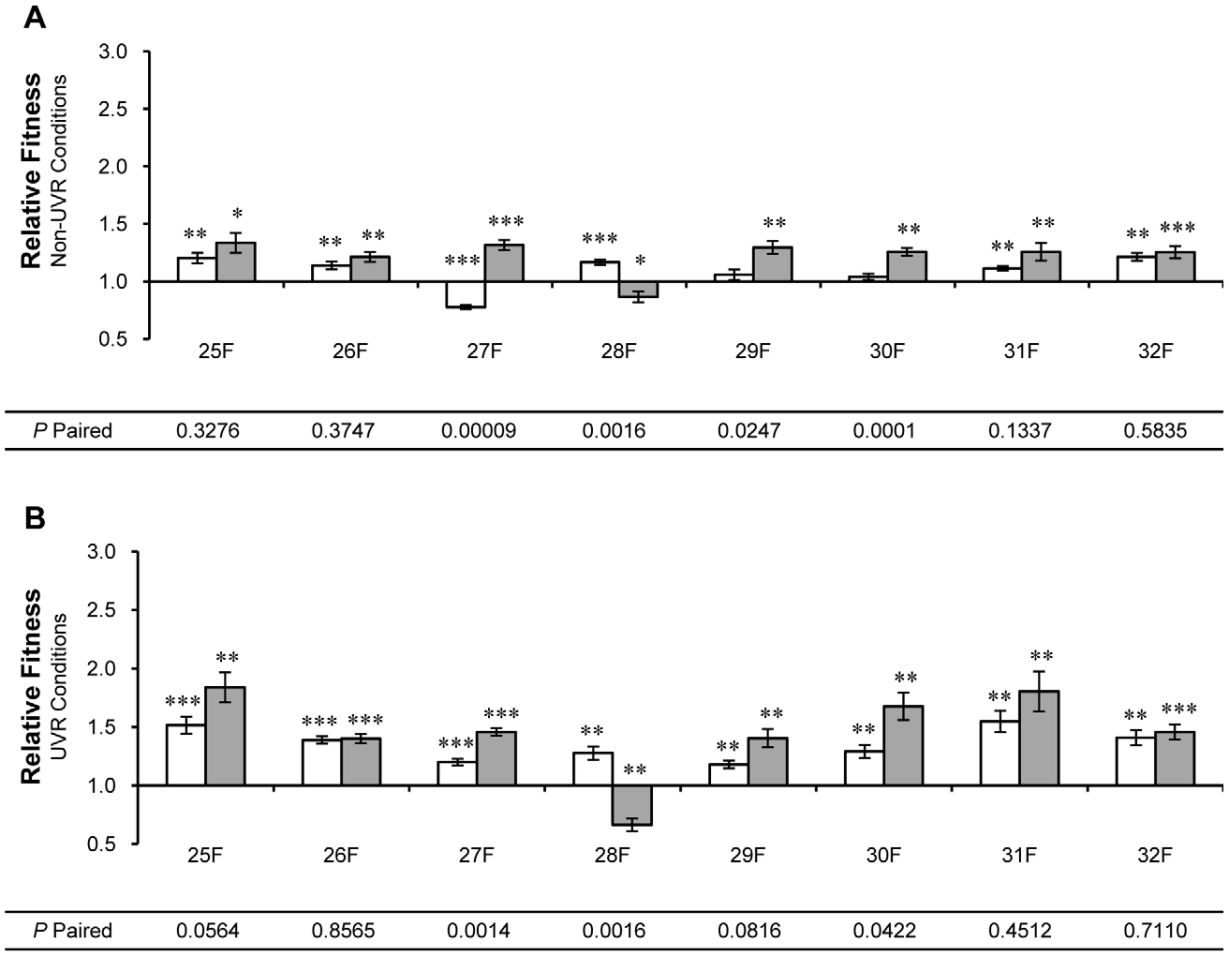
Relative fitness of ‘fuzzy’ (F) isolates from UVR lineages 25-32. Relative fitness was measured by direct competition with the ancestor under (A) non-UVR and (B) UVR conditions. Single isolates of *P. cichorii* 302959 exhibiting the ‘fuzzy’ colony morphology were taken after 250 (open bars) and 500 (shaded bars) generations of experimental evolution. Fitness values are means and error bars represent standard error of the mean. Values are significant by two-tailed independent *t*-test (d.f. = 5, α = 0.05) where indicated (* *P*<0.05, ** *P*<0.01, *** *P*<0.001). *P*-paired values correspond to two-tailed paired *t*-tests (d.f. = 5, α = 0.05) between relative fitness values for isolates from generation 250 and 500.

**Figure 6:**
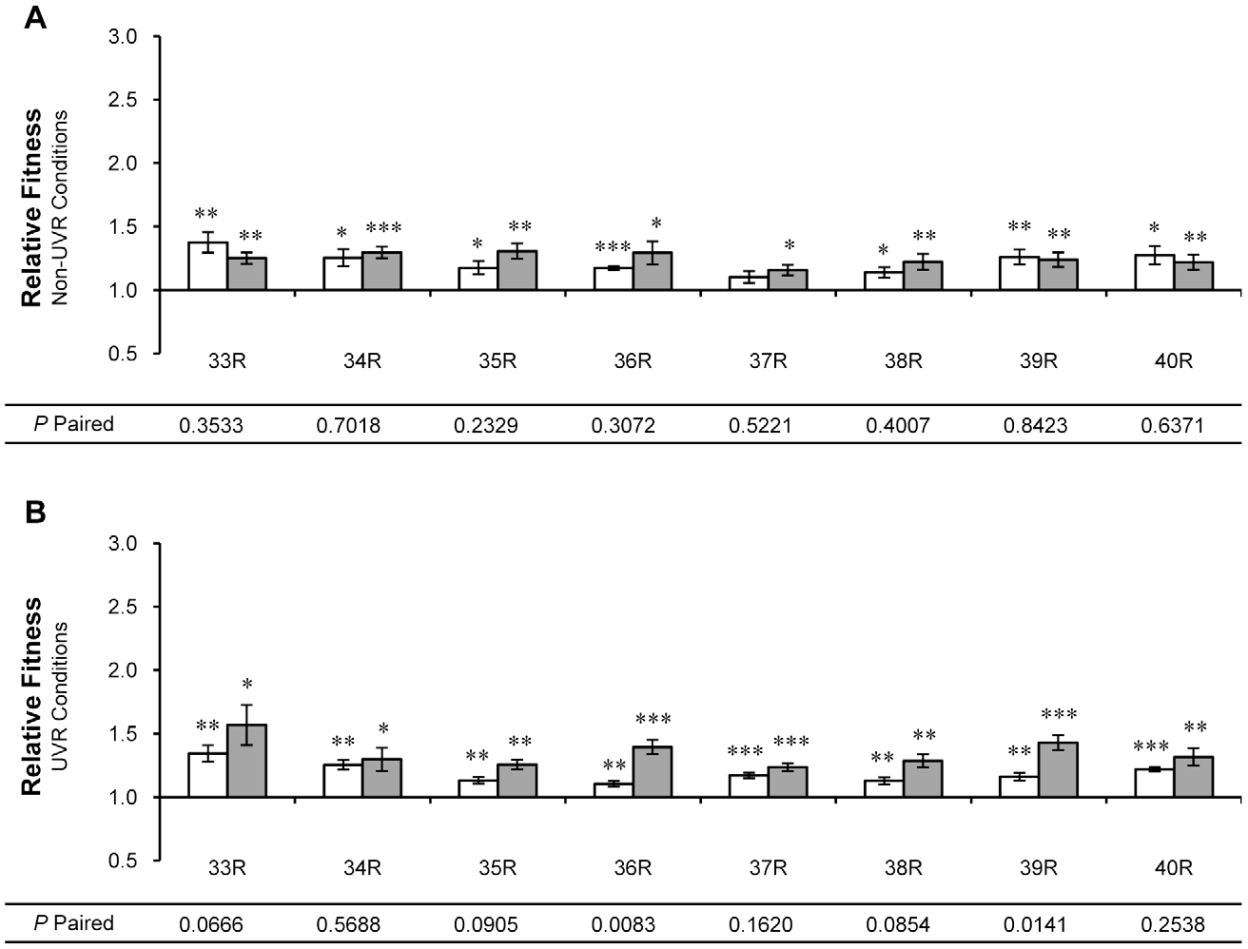
Relative fitness of isolates from non-UVR lineages 33-40. Relative fitness was measured by direct competition with the ancestor under (A) non-UVR and (B) UVR conditions. Non-UVR lineages contained only colonies with the ‘round’ morphology and single isolates of *P. cichorii* 302959 were taken after 250 (open bars) and 500 (shaded bars) generations of experimental evolution. Fitness values are means and error bars represent standard error of the mean. Values are significant by two-tailed independent *t*-test (d.f. = 5, α = 0.05) where indicated (* *P*<0.05, ** *P*<0.01, *** *P*<0.001). *P*-paired values correspond to two-tailed paired *t*-tests (d.f. = 5, α = 0.05) between relative fitness values for isolates from generation 250 and 500.

Isolates from UVR lineages expressed two different morphologies, R and F, and single-colony representatives of each from generations 250 and 500 were characterized. Neither morphotype group exhibited a significant fitness advantage under either UVR or non-UVR conditions (all *P*>0.320). Therefore, measurements of the two groups have been combined when comparing isolates from UVR and non-UVR lineages but are also presented individually to provide additional sampling of the genetic diversity present in UVR lineages. Relative fitness measurements were also conducted using population samples to observe any influence of genotype community structure in UVR lineages. We have combined the relative fitness values determined at generation 250 in this study with previously published measurements at generation 500 [12] in our data analysis and illustrations to highlight differences in adaptive improvements at the two time points. The average relative fitness of all isolates and population samples is summarized in Figure 2 and individual measurements of each lineage are reported in Figures 3, 4, 5, 6. Each relative fitness change was analyzed by an independent *t*-test against the hypothesis of mean fitness equal to that of the ancestral *P. cichorii* 302959 (*W* = 1) and is significant where indicated in the figures. Paired *t-*tests were also used to determine significant differences in relative fitness between growth environments (UVR vs. non-UVR conditions) or growth intervals (generations 250 vs. 500) and these *P* values are listed at the bottom of each figure.

Population samples of ∼10^8^ cells from UVR lineages at generation 250 exhibited increased relative fitness under non-UVR conditions and further improvements under UVR conditions (Figure 2A). Likewise, single-colony isolates from these UVR lineage communities exhibited increased relative fitness at generation 250 in favor of the UVR conditions (Figure 2A). Similar adaptive specificity was observable in population samples and isolates from UVR lineages at generation 500 (all *P*<0.0013, Figure 2B). The specific differences in relative fitness exhibited by UVR lineages under the two conditions were significant at both generations 250 and 500. Conversely, isolates from non-UVR lineages generally displayed comparable fitness gains under both non-UVR and UVR conditions (Figure 2A). The average relative fitness of population samples and isolates from all lineages at generation 500 was increased under UVR conditions compared to generation 250 (Figure 2B).

Population samples taken from individual UVR lineages at generation 250, with the exception of lineage 28, displayed a 15% average improvement in relative fitness under non-UVR conditions (all *P*<0.026, Figure 3A). Under UVR conditions, individual population samples from UVR lineages at generation 250 exhibited a 61% average improvement in relative fitness (all *P*<0.002, Figure 3B). Only population samples from lineages 28 and 32 exhibited significant differences in relative fitness under non-UVR conditions between generations 250 and 500 (all *P*<0.017, Figure 3A). However, relative fitness under UVR conditions exhibited by population samples from half of the UVR lineages differed significantly between generations 250 and 500 (all *P*<0.002, Figure 3B).

As described previously, the ancestral *P. cichorii* 302959 displayed a ‘round’ colony morphology that gave rise to complex mixtures of R and F morphotypes during the experimental evolution of all UVR lineages [12]. Under non-UVR conditions, individual R morphotype isolates recovered at generation 250 from only three UVR lineages (25, 26, and 27) exhibited improvements in relative fitness (all *P*<0.039, Figure 4A). However, all R isolates from UVR lineages at generation 250 gained relative fitness under UVR conditions by an average of 30% (all *P*<0.021, Figure 4B). Only an R morphotype isolated from lineage 28 at generation 250 displayed reduced fitness under non-UVR conditions (*P* = 0.004, Figure 4A). When compared to isolates taken at generation 500, only the relative fitness of R morphotype isolates from UVR lineages 30 and 32 differed significantly under non-UVR conditions (all *P*<0.02, Figure 4A). Under UVR conditions, the relative fitness of R morphotypes isolated from lineages 30, 31, and 32 differed significantly between generations 250 and 500 (all *P*<0.040, Figure 4B).

Nearly all F morphotypes isolated from UVR lineages at generation 250 exhibited increased fitness under non-UVR conditions by an average of 17% (all *P*<0.010, Figure 5A). Only an F morphotype isolated from lineage 27 at generation 250 exhibited reduced fitness under non-UVR conditions (*P* = 0.00005, Figure 5A). Under UVR conditions, all F isolates from UVR lineages at generation 250 gained relative fitness by an average of 35% (all *P*<0.005, Figure 5B). When compared to isolates taken at generation 500, the relative fitness of F morphotype isolates from UVR lineages 27, 28, 29 and 30 all differed significantly under non-UVR conditions (all *P*<0.025, Figure 5A). Under UVR conditions, the relative fitness of F morphotypes isolated from lineages 27, 28, and 30 differed significantly between generations 250 and 500 (all *P*<0.043, Figure 5B).

Non-UVR lineages 33–40 contained only R morphotypes. Single-colony isolates from generation 250 displayed a 24% average improvement in relative fitness under non-UVR conditions (all *P*<0.020) with the exception of the isolate from lineage 37 (Figure 6A). Isolates from non-UVR lineages at generation 250 also exhibited a 19% average improvement in relative fitness under UVR conditions (all *P*<0.006, Figure 6B). Only the relative fitness of the isolate from lineage 36 varied significantly between the two conditions (*P* = 0.029). When compared to isolates from generation 500, there was no significant difference in relative fitness under non-UVR conditions (Figure 6A) and only isolates from non-UVR lineages 36 and 39 differed significantly from their generation 500 counterparts under UVR conditions (Figure 5B).

### Growth dynamics

**Figure 7:**
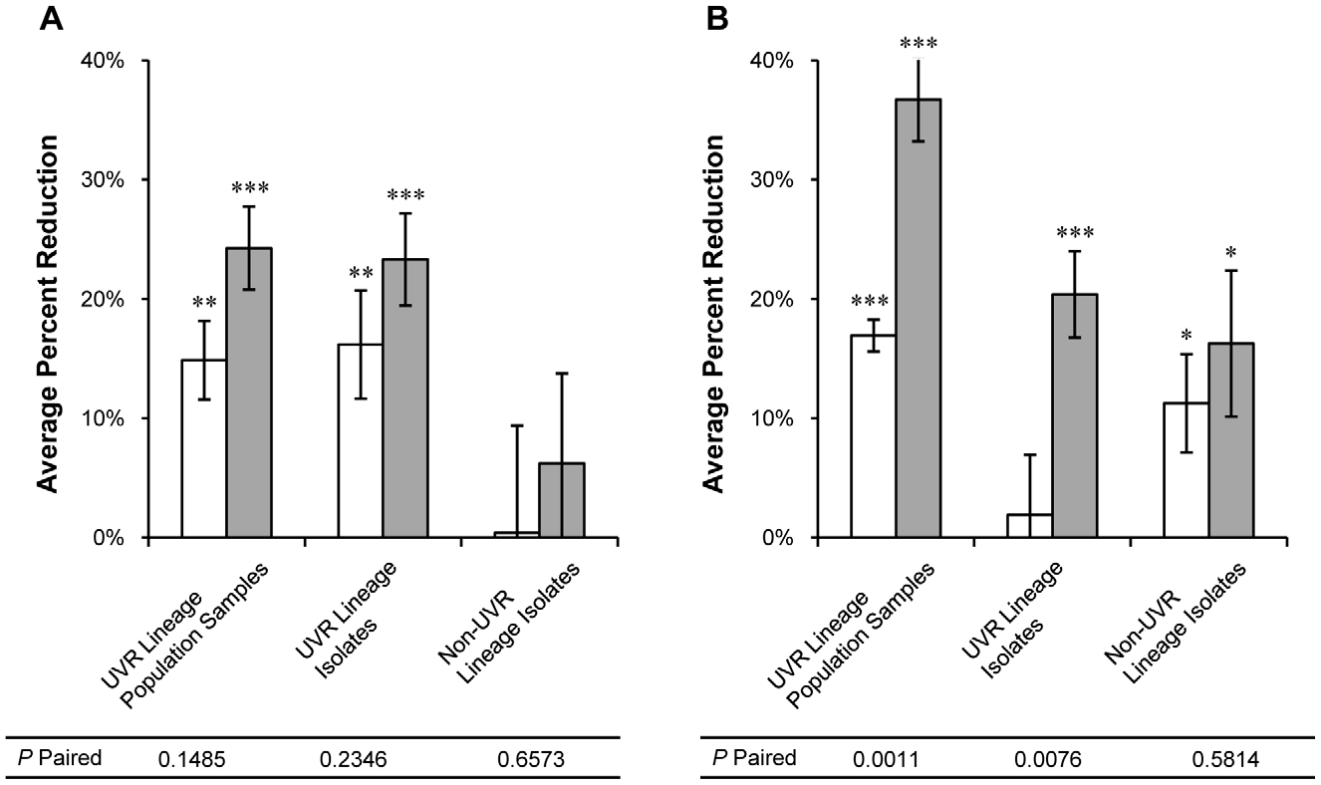
Average percent reduction in doubling time. Doubling time of UVR lineage population samples, UVR lineage isolates, and non-UVR lineage isolates after (A) 250 and (B) 500 generations of experimental evolution was measured under non-UVR (open bars) and UVR (shaded bars) conditions. Values represent the average percent improvement compared to the *P. cichorii* 302959 ancestor doubling times of 1.45±0.09 hrs and 1.68±0.10 hrs under non-UVR and UVR conditions, respectively. Error bars represent standard error of the mean. n = 8 for UVR lineage population samples and non-UVR lineage isolates but n = 16 for UVR lineage isolates. Values are significant by two-tailed independent *t*-test (α = 0.05) where indicated (* *P*<0.05, ** *P*<0.01, *** *P*<0.001). *P*-paired values correspond to two-tailed paired *t*-tests (α = 0.05) between non-UVR and UVR conditions.

Growth parameters were examined to elucidate components of adaptation observed in evolved isolates and population samples in an attempt to determine mechanisms responsible for specific changes in relative fitness. Growth curves of each population sample and single-colony isolate from generations 250 and 500 were plotted to calculate changes in doubling and lag times under both non-UVR and UVR conditions. Isolates and population samples from all evolved lineages displayed improvements in both doubling and lag times. The ancestral *P. cichorii* 302959 strain exhibited doubling times of 1.45±0.09 hours and 1.68±0.10 hours at 28°C under non-UVR and UVR conditions, respectively. Population samples from UVR lineages improved their doubling time under non-UVR conditions by an average reduction of 15% at generation 250 and 17% at generation 500 (Figure 7A and B). Doubling times of population samples were further reduced under UVR conditions by an average of 24% and 37% at generations 250 and 500, respectively (Figure 7A and B). Isolates from UVR lineages, including both R and F morphotypes, exhibited similar patterns of doubling time improvement, with greater average reductions under UVR conditions at both generation 250 and 500 (Figure 7A and B). However, differences in doubling time improvements under the two conditions were only significant at generation 500 for both population samples and isolates from UVR lineages. Conversely, the average doubling time of isolates from non-UVR lineages was more analogous under non-UVR and UVR conditions and reductions were only significant at generation 500 (Figure 7A and B).

**Figure 8:**
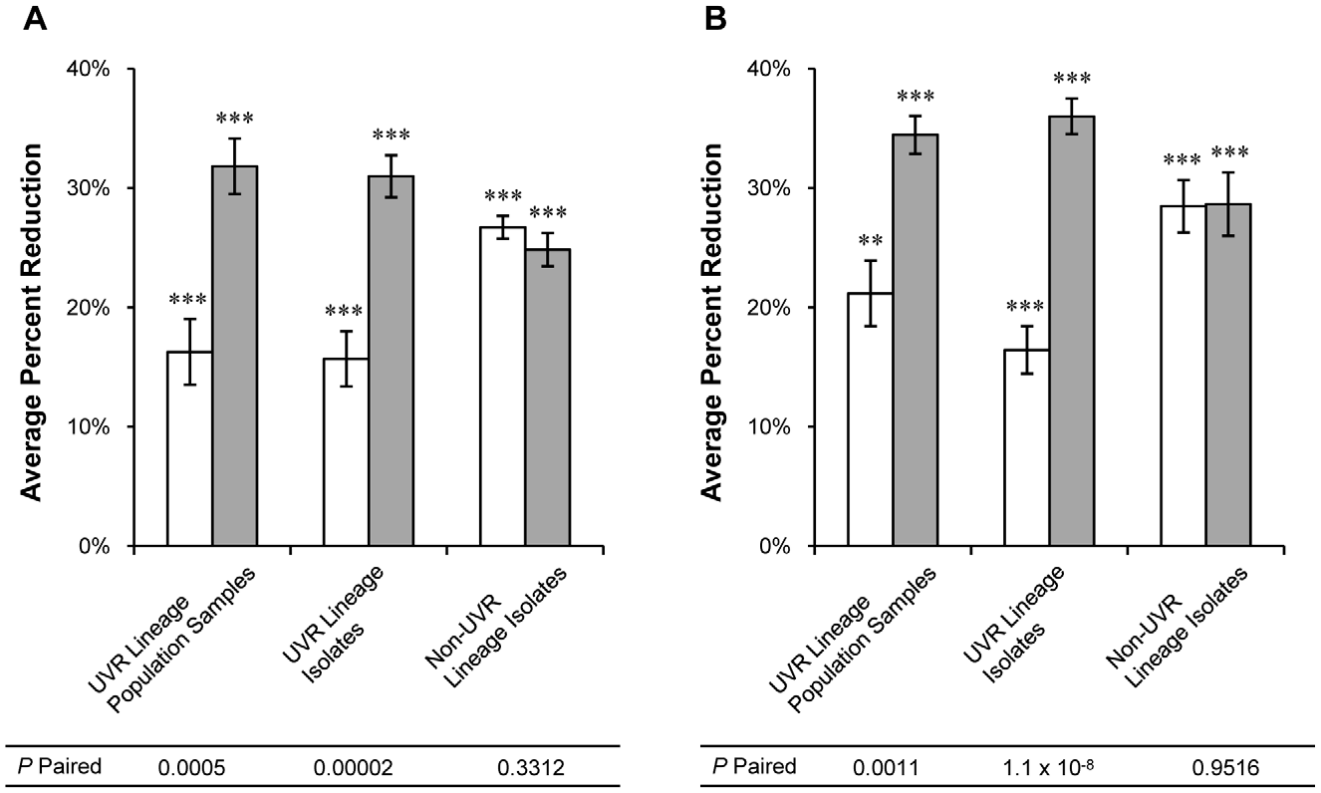
Average percent reduction in lag time. Lag time of UVR lineage population samples, UVR lineage isolates, and non-UVR lineage isolates after (A) 250 and (B) 500 generations of experimental evolution was measured under non-UVR (open bars) and UVR (shaded bars) conditions. Values represent the average percent improvement compared to the *P. cichorii* 302959 ancestor doubling times of 10.27±0.32 hrs and 10.47±0.26 hrs under non-UVR and UVR conditions, respectively. Error bars represent standard error of the mean. n = 8 for UVR lineage population samples and non-UVR lineage isolates but n = 16 for UVR lineage isolates. Values are significant by two-tailed independent *t*-test (α = 0.05) where indicated (* *P*<0.05, ** *P*<0.01, *** *P*<0.001). *P*-paired values correspond to two-tailed paired *t*-tests (α = 0.05) between non-UVR and UVR conditions.

Similar improvements in lag time (i.e. reductions in lag time) were also observed. The ancestral *P. cichorii* 302959 strain exhibited analogous lag times of 10.27±0.32 hours and 10.47±0.26 hours under non-UVR and UVR conditions, respectively. Population samples from UVR lineages improved their lag times under non-UVR conditions by an average reduction of 16% at generation 250 and 21% at generation 500 (Figure 8A and B). Lag times of population samples were further reduced under UVR conditions by an average of 32% at generation 250 and 34% at generation 500 (Figure 8A and B). The lag times of isolates from UVR lineages again exhibited similar patterns of improvement significantly with greater average reductions under UVR conditions at both generation 250 and 500 (Figure 8A and B). Conversely, isolates from non-UVR lineages exhibited comparable reductions in lag times under non-UVR and UVR conditions at both generation 250 and 500 (Figure 8A and B). A complete list of doubling and lag times for each population sample and single-colony isolate is available in Tables S1 and S2.

### UVR Tolerance

Changes in UVR tolerance could lead to changes in relative fitness under UVR conditions. Therefore, the UVR tolerance of population samples and isolates from each UVR and non-UVR lineages taken at generations 250 and 500 was determined following a single dose of UVC. An elevated dose of ∼140 J m^−2^ s^−1^ was used to resolve subtle differences in UVR tolerance that could not be distinguished at the lower dosage used during lineage propagation. The ancestral *P. cichorii* 302959 strain exhibited a 0.7% survival rate at the elevated dose. Population samples from UVR lineages displayed the most consistent improvements in UVR tolerance with average survival rates of 4.3% and 4.0% at generations 250 and 500, respectively. R and F morphotype isolates exhibited a mixture of UVR tolerance phenotypes that were higher, lower, or comparable to that of the ancestor. A complete list of UVR tolerance measures for each population sample and isolate is available in Table S3.

### Relative fitness trajectories

**Figure 9:**
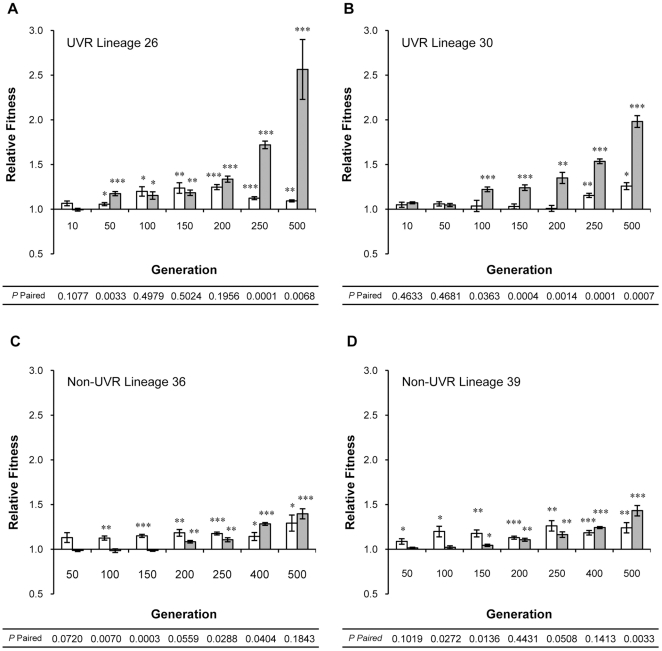
Relative fitness trajectories of population samples from select lineages of *P. cichorii* 302959. Relative fitness of population samples containing ∼10^8^ cells from UVR lineages (A) 26 and (B) 30 and non-UVR lineages (C) 26 and (D) 39 under non-UVR (open bars) and UVR (shaded bars) conditions was measured by direct competition with the ancestor. Fitness values are means and error bars represent standard error of the mean. Values are significant by two-tailed independent *t*-test (d.f. = 5, α = 0.05) where indicated (* *P*<0.05, ** *P*<0.01, *** *P*<0.001). *P*-paired values correspond to two-tailed paired *t*-tests (d.f. = 5, α = 0.05) between non-UVR and UVR relative fitness values.

To track changes in relative fitness during lineage growth over the course of 500 generations, preserved population samples from four randomly selected lineages were obtained at different intervals and competed against the *P. cichorii* 302959 ancestor under both non-UVR and UVR conditions. The resulting fitness trajectories are plotted in Figure 8. UVR lineages 26 and 30 first exhibited fitness gains under their native, UVR conditions before later gaining fitness under the alternate, non-UVR conditions (Figure 9A and B). The relative fitness of UVR lineage 26 remained comparable under both non-UVR and UVR conditions until additional fitness gains were later achieved under UVR conditions by generation 250 (Figure 9A). UVR lineage 30 displayed improved fitness only under UVR conditions for a much longer period until later gaining fitness under non-UVR conditions (Figure 9B). However, even after gaining fitness under non-UVR conditions, UVR lineage 30 continued to exhibit significantly higher fitness under UVR conditions.

Similarly, non-UVR lineages 36 and 39 exhibited fitness gains under their native, non-UVR conditions first (Fig. 9C and D). Neither lineage 36 nor 39 exhibited a significant increase in non-UVR fitness following the initial improvements observed in the first 100 generations. However, additional fitness improvements under UVR conditions were achieved before generation 500 in both non-UVR lineages examined (Figure 9C and D).

## Discussion

Our results suggest that adaptive specificity in laboratory evolved populations of the highly UVR-mutable *P. cichorii* 302959 emerged early and correlated with discrete growth improvements but was not dependent upon MDR activation. In a previous study, we maintained parallel cultures of *P. cichorii* 302959 for 500 generations with daily MDR activation in the form of UVR exposure and observed higher relative fitness particularly under UVR conditions [12]. In the present study, changes in relative fitness and underlying physiological characteristics were analyzed at intermediate points (in particular, at generation 250) in the experimental evolution of *P. cichorii* 302959 to evaluate this observed adaptive specificity.

The relative fitness of all 16 lineages was determined at the generation 250 midpoint of our evolution experiment with the intent to gain further information on the acquisition of fitness observed previously at the generation 500 endpoint. Fitness measurements at the midpoint can offer some indication of the timing, number, magnitude, and parallel nature of adaptive improvements achieved by each lineage. Measurements of relative fitness at generation 250 reflected the same patterns of adaptive specificity observed previously at generation 500 [12]. The interaction between sympatric genotypes within UVR lineages was previously shown to contribute to relative fitness under UVR conditions [12]. Such ecological interactions were established in UVR lineages by generation 250 and population samples exhibited greater fitness gains than individual isolates. A comparison of relative fitness values at different time points indicated that most relative fitness improvements observed in *P. cichorii* 302959 lineages emerged within the first 250 generations of growth. Additional fitness gains attained between generations 250 and 500 were primarily achieved under UVR conditions, even in non-UVR lineages. The dramatic changes in fitness exhibited by F isolates from lineages 27 and 28 demonstrate that UVR lineage populations contained a dynamic balance of genotypes, and that isolates sampled at generation 500 were not necessarily derived from those sampled at generation 250. Together, these results emphasize the complexity of adaptation in the UVR lineage populations due to the high adaptive potential and plasticity of the ancestral *P. cichorii* 302959 inducible mutator genotype. Additionally, the increase in relative fitness observed only under UVR conditions between generations 250 and 500 in some non-UVR lineages offers the first indication that beneficial mutations differentially contribute to fitness under the two growth conditions.

Measures of relative fitness indicate the overall advantage of one bacterial strain or population over another but provide no information about the underlying physiological differences responsible for the advantage. Changes in growth dynamics have been identified as distinct components of relative fitness improvements in similar studies conducted previously [22], [23]. We plotted optical density changes during growth of isolates and population samples from lineages of *P. cichorii* 302959 obtained at generations 250 and 500 under both UVR and non-UVR conditions. Growth dynamic improvements correlated well with changes in relative fitness such that nearly all evolved lineages with increased fitness displayed corresponding reductions in both doubling and lag times compared to the ancestor. The average reductions in both lag and doubling times exhibited by UVR lineages were greatest under UVR conditions while non-UVR lineages displayed comparable reductions under both conditions. These results are consistent with the observed patterns of adaptive specificity in relative fitness measurements suggesting that specific growth improvements are responsible for the characteristic gains in relative fitness displayed by UVR lineages under UVR conditions.

Improvements in lag time could be indicative of a faster metabolic transition from stationary phase to exponential growth due to adaptation to the culture media or to the cyclic nature of the serial transfer regime. Improvements in lag time under UVR conditions specifically could result from increased UVR tolerance or more efficient DNA repair in the form of improved SOS induction or polymerase processivity. The complex SOS regulatory network is comprised of both transcriptional and post-transcriptional regulators that tightly control the temporal activity of its gene products [8], [24], [25]. Beneficial adaptations targeted to any of these regulators that contribute to the timing or efficiency of the SOS network could be observable as a reduction in lag time particularly under UVR conditions.

We explored changes in UVR tolerance as an obvious candidate for improved fitness and lag time under UVR conditions but only observed increased UVR tolerance concurrently with fitness gains in a limited number of instances. In general, there was no pattern to suggest that greater UVR tolerance was primarily responsible for increased relative fitness observed under UVR conditions. Alternatively, improved fitness under UVR conditions could result from enhanced nutrient scavenging. Approximately 90% of the population died as a result of UVR exposure during each cycle of the UVR lineage regime. The death of these individuals could release additional nutrients in the form of cell lysate that is transferred with surviving cells to fresh medium for the next cycle of growth. Any mutation that would enhance the utilization of these nutrients could lead to greater fitness exclusively under UVR conditions. In evolution experiments with *E. coli*, adaptive mutations that contribute to survival through catabolism of cellular detritus [26] or secreted metabolites [27] have been reported. However, we were unable to observe growth by the ancestral *P. cichorii* 302959 or any evolved lineages in glucose-free Davis Minimal medium amended with a bacterial suspension killed either by prolonged heat or UVR exposure (data not shown). Therefore, increased relative fitness under UVR conditions and the adaptive specificity of UVR lineages that is reflected in improved growth likely did not result from either increased UVR tolerance or a heightened ability to scavenge nutrients as a carbon source.

We previously observed the reproducible emergence of an F morphotype that coexisted with the ancestral, R morphotype in all UVR lineages but never in non-UVR lineages [12]. In the current study, single-colony representatives of both morphologies have been examined for changes in relative fitness and physiology at generation 250 and 500 under both UVR and non-UVR conditions. We have found no evidence to suggest that either morphotype possesses an inherent adaptive advantage observable as improved relative fitness, growth dynamics, or UVR tolerance. In our analyses we found only subtle differences in doubling times between the two groups. The results of the current study are consistent with our previous observations of the stochastic fluctuations of F morphotype abundance [12] suggesting the F determinant itself likely does not carry a strong selective advantage. Therefore, measurements of the two groups can be combined and interpreted as additional sampling of the genetic diversity present in the UVR lineages.

It is clear from our relative fitness measurements that adaptive changes in both non-UVR and UVR lineages were concentrated in the first 250 generations, confirming both the strong selection pressure of the experimental environments and the high adaptive potential of the ancestral *P. cichorii* 302959 genotype. Furthermore, the early fitness improvements by UVR lineages are consistent with the emergence of F morphotypes and spontaneous rifampicin-resistant colonies reported previously [12]. For a more detailed view of adaptation, we tracked the acquisition of relative fitness under UVR and non-UVR conditions in four randomly selected lineages. The resulting fitness trajectories are somewhat punctuated, suggesting adaptive evolution due to the successive acquisition of beneficial mutations consistent with similar studies conducted previously [28]. Rapid changes in relative fitness interrupted periods of apparent stasis throughout the history of each lineage. However, these populations remained quite dynamic, as the frequency of F morphotypes and rifampicin-resistant colonies continued to fluctuate [12] without affecting the overall fitness of the population. The fitness trajectories in this study confirm that within communities assembled by adaptive radiation, such as those present in our UVR lineages, changes in individual fitness produce fluctuations in genotype frequency, and the interactions of those sympatric genotypes influence the relative fitness of the population [29]. Furthermore, complete selective sweeps are not required for adaptive improvements to influence the fitness of a population.

The fitness trajectories in this study also suggest that adaptive improvements differentially contributed to relative fitness gains under UVR and non-UVR conditions. The ordered acquisition of improvements in the two environments favored the respective conditions under which each lineage was propagated such that UVR lineages first exhibited specific fitness gains under UVR conditions and non-UVR lineages under non-UVR conditions. Based on our previous observations of relative fitness at generation 500, we concluded that non-UVR lineages exhibited comparable fitness gains under UVR and non-UVR conditions due to their adaptation to the shared culture medium [12]. We expected that any phenotypic traits contributing to success under non-UVR conditions should likewise be beneficial during growth under UVR conditions. However, it is clear from fitness trajectories of non-UVR lineages that increases in fitness under non-UVR conditions were not mirrored by comparable increases in fitness under UVR conditions. These results suggest that adaptive specificity does not depend on MDR activation but is in fact a common pattern of adaption in *P. cichorii* 302959 exhibited by both UVR and non-UVR lineages. Such adaptive specialization can result in fitness trade-offs due to either antagonistic pleiotropy or mutation accumulation resulting in lower fitness under different environmental conditions [15], [16], [30]. However, in this study the adaptive specificity of both UVR and non-UVR lineages did not include any observable negative effect on fitness in the respective alternate environments.

Interestingly, when fitness did increase in the alternate environment, corresponding improvements in fitness were not observed in the native environment. It is unlikely that fitness improvements under UVR and non-UVR conditions are independent. Rather, mutations that contribute to fitness observable only in the alternate environment may represent general improvements of smaller effect in the native environment. After gaining fitness in the alternate environment, UVR lineages continued to gain fitness specifically under their native conditions while non-UVR lineages did not, producing the illusion of adaptive specificity exclusively in UVR lineages. We can propose three possible explanations for this discrepancy: (1) MDR activation provides access to more beneficial mutations by altering the mutation spectrum of UVR lineages; (2) by increasing the overall mutation rate, MDR activation increases the rate at which beneficial mutations appear in the population and non-UVR lineages would gain additional relative fitness if propagated for a longer time; or (3) the ancestral *P. cichorii* 302959 genotype is more poorly adapted to the UVR conditions and therefore more adaptive opportunities exist than under non-UVR conditions. Although the first two possibilities lend support to the proposed positive influence of inducible mutability on adaptive evolution, we do not have sufficient information to distinguish them from each other or from the third possibility.

In summary, we have investigated the adaptive specificity of *P. cichorii* 302959 during experimental evolution by measuring relative fitness and corresponding changes in physiology. Our results suggest that adaptive specificity in this organism correlates with discrete growth improvements but does not depend on MDR activation. Adaptive improvements in all lineages were concentrated in the first 250 generations of experimental evolution and specific increases in relative fitness correlated with distinct improvements in doubling and lag times. Furthermore, UVR lineages exhibited additional gains in fitness after generation 250 exclusively under UVR conditions that were reflected in further doubling and lag time improvements but likely not greater UVR tolerance or scavenging of nutrients to support the growth of new biomass. Fitness trajectories of select lineages clearly indicate that adaptive improvements under UVR and non-UVR conditions were acquired preferentially and differentially contributed to relative fitness under varied growth conditions.

The results of this study lend support to our earlier observations that suggest increased mutation rate in the form of inducible mutability does not impede adaptation by mutation accumulation. Rather, UVR and non-UVR lineages preferentially acquired adaptive growth improvements in a similar manner and additional fitness gains by UVR lineages may have been due to greater access to beneficial mutations. Our future work will compare the influence of different mechanisms of mutability on adaptation in an advanced genetic system equipped with genomic tools for analysis.

## Supporting Information

Table S1 Doubling time of population samples and isolates from lineages of *P. cichorii* 302959 during growth under non-UVR and UVR conditions.
(DOCX)

Table S2 Lag time of population samples and isolates from lineages of *P. cichorii* 302959 during growth under non-UVR and UVR conditions.
(DOCX)

Table S3 Percent survival of population samples and isolates from lineages of *P. cichorii* 302959 following ∼140 J m^−2^ UVC.
(DOCX)

## References

[1] De Visser JAGM, Rozen DE (2005). Limits to adaptation in asexual populations.. J Evol Biol.

[2] De Visser JAGM (2002). The fate of microbial mutators.. Microbiology.

[3] Sundin GW, Weigand MR (2007). The microbiology of mutability.. FEMS Microbiol Lett.

[4] Hall LMC, Henderson-Begg SK (2006). Hypermutable bacteria isolated from humans – a critical analysis.. Microbiology.

[5] Giraud A, Radman M, Matic I, Taddei R (2001). The rise and fall of mutator bacteria.. Curr Opin Microbiol.

[6] Tenaillon O, Taddei F, Radman M, Matic I (2001). Second order selection in bacterial evolution: selection acting on mutation and recombination rates in the course of adaptation.. Res Microbiol.

[7] Schlacher K, Goodman MF (2007). Lessons from 50 years of SOS DNA-damage-induced mutagenesis.. Nat Rev Mol Cell Biol.

[8] Courcelle J, Khodursky A, Peter B, Brown PO, Hanawalt PC (2001). Comparative gene expression profiles following UV exposure in wild-type and SOS-deficient *Escherichia coli*.. Genetics.

[9] Jarosz DF, Beuning PJ, Cohen SE, Walker GC (2007). Y-family DNA polymerases in *Escherichia coli*.. Trends Microbiol.

[10] Kim JJ, Sundin GW (2000). Regulation of the *rulAB* mutagenic DNA repair operon of *Pseudomonas syringae* by UV-B (290 to 320 nanometers) radiation and analysis of *rulAB*-mediated mutability *in vitro* and *in planta*.. J Bacteriol.

[11] Sundin GW, Murillo J (1999). Functional analysis of the *Pseudomonas syringae rulAB* determinant in tolerance to ultraviolet B (290–320 nm) radiation and distribution of *rulAB* among *P. syringae* pathovars.. Environ Microbiol.

[12] Weigand MR, Sundin GW (2009). Long-term effects of inducible mutagenic DNA repair on relative fitness and phenotypic diversification in *Pseudomonas cichorii* 302959.. Genetics.

[13] Zhang S, Sundin GW (2004). Mutagenic DNA repair potential in *Pseudomonas* spp., and characterization of the *rulAB*
_Pc_ operon from the highly mutable strain *Pseudomonas cichorii* 302959.. Can J Microbiol.

[14] Loh E, Salk JJ, Loeb LA (2010). Optimization of DNA polymerase mutation rates during bacterial evolution.. Proc Natl Acad Sci USA.

[15] MacLean RC, Bell G, Rainey PB (2004). The evolution of a pleiotropic fitness tradeoff in *Pseudomonas fluorescens*.. Proc Natl Acad Sci USA.

[16] Cooper VS, Lenski RE (2000). The population genetics of ecological specialization in evolving *Escherichia coli* populations.. Nature.

[17] King EO, Ward MK, Raney DC (1954). Two simple media for the demonstration of pyocyanin and fluorescin.. J Lab Clin Med.

[18] Choi KH, Kumar A, Scheizer HP (2006). A 10-min method for preparation of highly electrocompetent *Pseudomonas aeruginosa* cells: application for DNA fragment transfer between chromosomes and plasmid transformation.. J Microbiol Meth.

[19] Sambrook J, Fritsch EF, Maniatis T (1989). Molecular Clonging: A Laboratory Manual..

[20] Lenski RE, Souza V, Duong LP, Phan QG, Nguyen TNM (1994). Epistatic effects of promoter and repressor functions of the Tn10 tetracycline-resistance operon on the fitness of *Escherichia coli*.. Mol Ecol.

[21] Madigan MT, Martinko JM (2006). Brock Biology of Microorganisms, 11^th^ Edition..

[22] Sleight SC, Lenski RE (2007). Evolutionary adaptation to freeze-thaw-growth cycles in *Escherichia coli*.. Physiol Biochem Zool.

[23] Lenski RE, Mongold JA, Sniegowski PD, Travisano M, Vasi F (1998). Evolution of competitive fitness in experimental populations of *E. coli*: What makes one genotype a better competitor than another?. Antonie van Leeuwenhoek.

[24] Shimoni Y, Altuvia S, Margalit H, Biham O (2009). Stochastic analysis of the SOS response in *Escherichia coli*.. PLoS ONE.

[25] Friedman N, Vardi S, Ronen M, Alon U, Stavans J (2005). Precise temporal modulation in the response of the SOS DNA repair network of individual bacteria.. PLoS Biol.

[26] Finkel SE (2006). Long-term survival during stationary phase: evolution and the GASP phenotype.. Nat Rev Microbiol.

[27] Turner PE, Souza V, Lenski RE (1996). Tests of ecological mechanisms promoting the stable coexistence of two bacterial genotypes.. Ecology.

[28] Elena SF, Cooper VS, Lenski RE (1996). Punctuated evolution caused by selection of rare beneficial mutations.. Science.

[29] MacLean RC (2005). Adaptive radiation in microbial microcosms.. J Evol Biol.

[30] Travisano M, Lenski RE (1996). Long-term experimental evolution in *Escherichia coli* IV. Targets of Selection and the specificity of adaptation.. Genetics.

