# Supplementary material for: Growth Parameter Components of Adaptive Specificity during Experimental Evolution of the UVR-Inducible Mutator Pseudomonas cichorii 302959
Source: PLoS One. 2011 Jan 14;6(1):e15975. doi: 10.1371/journal.pone.0015975 (PMC3021522; doi:10.1371/journal.pone.0015975)
Supplement: Table S1 — Doubling time of population samples and isolates from lineages of P. cichorii 302959 during growth under non-UVR and UVR conditions. (DOCX) [file pone.0015975.s001.docx]

TABLE S1. Doubling time (in hrs) of population samples and isolates from lineages of *P. cichorii* 302959 under non-UVR and UVR conditions.^a^

|  | Generation 250 | |  | Generation 500 | |
| --- | --- | --- | --- | --- | --- |
|  | Non-UVR | UVR |  | Non-UVR | UVR |
| UVR Lineage Population Samples | | |  |  |  |
| 25 | 1.10 ± 0.06 | 1.32 ± 0.07 |  | 1.25 ± 0.30 | 1.04 ± 0.04 |
| 26 | 0.94 ± 0.08 | 1.60 ± 0.19 |  | 1.19 ± 0.10 | 0.94 ± 0.08 |
| 27 | 1.11 ± 0.14 | 1.30 ± 0.03 |  | 1.20 ± 0.15 | 1.11 ± 0.14 |
| 28 | 0.98 ± 0.14 | 1.04 ± 0.05 |  | 1.09 ± 0.11 | 0.98 ± 0.14 |
| 29 | 0.96 ± 0.25 | 1.24 ± 0.05 |  | 1.20 ± 0.07 | 0.96 ± 0.25 |
| 30 | 0.86 ± 0.14 | 1.26 ± 0.13 |  | 1.26 ± 0.18 | 0.86 ± 0.14 |
| 31 | 1.23 ± 0.10 | 1.12 ± 0.05 |  | 1.26 ± 0.16 | 1.23 ± 0.10 |
| 32 | 1.37 ± 0.15 | 1.27 ± 0.17 |  | 1.19 ± 0.12 | 1.37 ± 0.15 |
| Round UVR Lineage Isolates | | |  |  |  |
| 25R | 1.18 ± 0.08 | 1.05 ± 0.30 |  | 1.58 ± 0.04 | 1.88 ± 0.27 |
| 26R | 1.19 ± 0.10 | 1.07 ± 0.06 |  | 1.65 ± 0.04 | 1.29 ± 0.12 |
| 27R | 1.28 ± 0.23 | 1.14 ± 0.09 |  | 2.32 ± 0.11 | 1.02 ± 0.17 |
| 28R | 0.89 ± 0.20 | 1.23 ± 0.12 |  | 1.61 ± 0.05 | 1.10 ± 0.23 |
| 29R | 1.05 ± 0.18 | 0.69 ± 0.20 |  | 1.40 ± 0.05 | 1.73 ± 0.32 |
| 30R | 1.13 ± 0.05 | 1.62 ± 0.23 |  | 1.07 ± 0.07 | 1.37 ± 0.04 |
| 31R | 1.25 ± 0.06 | 1.18 ± 0.13 |  | 1.42 ± 0.08 | 1.68 ± 0.10 |
| 32R | 1.93 ± 0.19 | 1.41 ± 0.07 |  | 1.12 ± 0.07 | 1.32 ± 0.07 |
| Fuzzy UVR Lineage Isolates | | |  |  |  |
| 25F | 1.34 ± 0.09 | 1.47 ± 0.33 |  | 1.19 ± 0.11 | 1.13 ± 0.16 |
| 26F | 1.33 ± 0.16 | 1.10 ± 0.03 |  | 1.57 ± 0.11 | 1.31 ± 0.11 |
| 27F | 1.09 ± 0.20 | 1.47 ± 0.20 |  | 1.17 ± 0.03 | 1.30 ± 0.11 |
| 28F | 1.10 ± 0.11 | 1.26 ± 0.04 |  | 1.47 ± 0.07 | 1.44 ± 0.21 |
| 29F | 1.24 ± 0.17 | 1.34 ± 0.05 |  | 1.34 ± 0.20 | 1.29 ± 0.31 |
| 30F | 1.59 ± 0.04 | 1.39 ± 0.25 |  | 1.44 ± 0.08 | 1.12 ± 0.08 |
| 31F | 0.84 ± 0.18 | 1.34 ± 0.19 |  | 1.55 ± 0.10 | 1.12 ± 0.11 |
| 32F | 1.00 ± 0.09 | 1.81 ± 0.13 |  | 1.43 ± 0.06 | 1.25 ± 0.10 |
| Non-UVR Lineage Isolates | | |  |  |  |
| 33R | 1.18 ± 0.07 | 1.62 ± 0.19 |  | 1.20 ± 0.11 | 1.44 ± 0.16 |
| 34R | 1.38 ± 0.26 | 1.84 ± 0.06 |  | 1.38 ± 0.02 | 1.39 ± 0.18 |
| 35R | 1.66 ± 0.08 | 1.94 ± 0.07 |  | 1.38 ± 0.07 | 1.58 ± 0.03 |
| 36R | 1.71 ± 0.14 | 1.13 ± 0.12 |  | 1.49 ± 0.04 | 1.21 ± 0.08 |
| 37R | 1.70 ± 0.15 | 1.51 ± 0.02 |  | 1.37 ± 0.03 | 1.69 ± 0.20 |
| 38R | 1.96 ± 0.08 | 1.45 ± 0.16 |  | 1.30 ± 0.06 | 1.03 ± 0.13 |
| 39R | 0.97 ± 0.23 | 2.03 ± 0.37 |  | 0.93 ± 0.09 | 1.12 ± 0.18 |
| 40R | 0.99 ± 0.13 | 1.06 ± 0.06 |  | 1.24 ± 0.03 | 1.07 ± 0.07 |

^a^ Doubling times of the *P. cichorii* 302959 ancestor were 1.45 ± 0.09 hrs and 1.68 ± 0.10 hrs under non-UVR and UVR conditions, respectively.
