# Supplementary material for: Growth Parameter Components of Adaptive Specificity during Experimental Evolution of the UVR-Inducible Mutator Pseudomonas cichorii 302959
Source: PLoS One. 2011 Jan 14;6(1):e15975. doi: 10.1371/journal.pone.0015975 (PMC3021522; doi:10.1371/journal.pone.0015975)
Supplement: Table S2 — Lag time of population samples and isolates from lineages of P. cichorii 302959 during growth under non-UVR and UVR conditions. (DOCX) [file pone.0015975.s002.docx]

TABLE S2. Lag time (in hrs) of population samples and isolates from lineages of *P. cichorii* 302959 under non-UVR and UVR conditions. ^a^

|  | Generation 250 | |  | Generation 500 | |
| --- | --- | --- | --- | --- | --- |
|  | Non-UVR | UVR |  | Non-UVR | UVR |
| UVR Lineage Population Samples | | |  |  |  |
| 25 | 9.83 ± 0.19 | 7.24 ± 0.09 |  | 6.86 ± 0.28 | 6.36 ± 0.04 |
| 26 | 8.18 ± 0.17 | 6.93 ± 0.20 |  | 8.20 ± 0.29 | 7.42 ± 0.05 |
| 27 | 8.43 ± 0.10 | 6.80 ± 0.07 |  | 7.46 ± 0.31 | 7.01 ± 0.16 |
| 28 | 9.85 ± 0.22 | 8.33 ± 0.09 |  | 9.48 ± 0.11 | 7.36 ± 0.17 |
| 29 | 8.16 ± 0.21 | 8.02 ± 0.10 |  | 8.62 ± 0.13 | 6.99 ± 0.21 |
| 30 | 8.53 ± 0.11 | 6.38 ± 0.21 |  | 7.63 ± 0.30 | 7.10 ± 0.20 |
| 31 | 8.04 ± 0.32 | 6.63 ± 0.06 |  | 8.45 ± 0.07 | 6.56 ± 0.11 |
| 32 | 7.76 ± 0.20 | 6.80 ± 0.25 |  | 8.05 ± 0.35 | 6.12 ± 0.06 |
| Round UVR Lineage Isolates | | |  |  |  |
| 25R | 10.01 ± 0.27 | 7.08 ± 0.23 |  | 8.83 ± 0.02 | 5.48 ± 0.23 |
| 26R | 9.19 ± 0.27 | 7.65 ± 0.25 |  | 10.24 ± 0.08 | 6.88 ± 0.08 |
| 27R | 7.78 ± 0.36 | 7.45 ± 0.34 |  | 9.60 ± 0.23 | 7.66 ± 0.14 |
| 28R | 10.04 ± 0.11 | 6.93 ± 0.10 |  | 8.26 ± 0.07 | 6.70 ± 0.10 |
| 29R | 9.94 ± 0.33 | 9.45 ± 0.24 |  | 8.14 ± 0.08 | 6.72 ± 0.35 |
| 30R | 7.74 ± 0.34 | 7.00 ± 0.22 |  | 7.70 ± 0.08 | 6.46 ± 0.09 |
| 31R | 7.83 ± 0.18 | 6.63 ± 0.19 |  | 8.25 ± 0.10 | 6.21 ± 0.16 |
| 32R | 7.74 ± 0.24 | 7.24 ± 0.29 |  | 7.59 ± 0.06 | 6.02 ± 0.10 |
| Fuzzy UVR Lineage Isolates | | |  |  |  |
| 25F | 8.55 ± 0.06 | 7.59 ± 0.05 |  | 7.62 ± 0.07 | 6.46 ± 0.32 |
| 26F | 7.41 ± 0.29 | 7.15 ± 0.29 |  | 8.79 ± 0.01 | 6.57 ± 0.20 |
| 27F | 9.86 ± 0.05 | 7.30 ± 0.25 |  | 8.17 ± 0.02 | 7.04 ± 0.16 |
| 28F | 9.48 ± 0.37 | 6.84 ± 0.09 |  | 10.23 ± 0.08 | 7.86 ± 0.04 |
| 29F | 8.64 ± 0.15 | 6.79 ± 0.13 |  | 8.44 ± 0.18 | 5.94 ± 0.12 |
| 30F | 7.89 ± 0.03 | 7.86 ± 0.03 |  | 8.37 ± 0.08 | 6.88 ± 0.32 |
| 31F | 8.38 ± 0.22 | 6.26 ± 0.16 |  | 8.79 ± 0.09 | 7.25 ± 0.17 |
| 32F | 8.05 ± 0.30 | 6.42 ± 0.02 |  | 8.27 ± 0.11 | 7.10 ± 0.16 |
| Non-UVR Lineage Isolates | | |  |  |  |
| 33R | 8.24 ± 0.44 | 7.28 ± 0.39 |  | 8.58 ± 0.07 | 7.80 ± 0.08 |
| 34R | 7.26 ± 0.15 | 7.64 ± 0.18 |  | 7.06 ± 0.14 | 7.24 ± 0.25 |
| 35R | 7.42 ± 0.05 | 7.75 ± 0.18 |  | 7.02 ± 0.09 | 6.92 ± 0.10 |
| 36R | 7.54 ± 0.23 | 7.99 ± 0.09 |  | 6.80 ± 0.34 | 7.45 ± 0.13 |
| 37R | 7.55 ± 0.25 | 8.47 ± 0.19 |  | 6.68 ± 0.06 | 6.55 ± 0.34 |
| 38R | 7.90 ± 0.17 | 8.20 ± 0.04 |  | 7.44 ± 0.25 | 6.96 ± 0.14 |
| 39R | 7.89 ± 0.12 | 7.43 ± 0.24 |  | 7.93 ± 0.07 | 7.75 ± 0.06 |
| 40R | 7.10 ± 0.23 | 8.19 ± 0.18 |  | 7.24 ± 0.11 | 9.10 ± 0.21 |

^a^ Lag times of the *P. cichorii* 302959 ancestor were 10.27 ± 0.32 hrs and 10.47 ± 0.26 hrs under non-UVR and UVR conditions, respectively.
