# Supplementary material for: Growth Parameter Components of Adaptive Specificity during Experimental Evolution of the UVR-Inducible Mutator Pseudomonas cichorii 302959
Source: PLoS One. 2011 Jan 14;6(1):e15975. doi: 10.1371/journal.pone.0015975 (PMC3021522; doi:10.1371/journal.pone.0015975)
Supplement: Table S3 — Percent survival of population samples and isolates from lineages of P. cichorii 302959 following ∼140 J m−2 UVC. (DOCX) [file pone.0015975.s003.docx]

TABLE S3. Percent survival of population samples and isolates from lineages of *P. cichorii* 302959 following ~140 J m^-2^ UVC. ^a^

|  | Gen 250 |  | Gen 500 |
| --- | --- | --- | --- |
| UVR Lineage Population Samples | | | |
| 25 | 6.1% |  | 5.0% |
| 26 | 5.0% |  | 1.5% |
| 27 | 6.0% |  | 0.4% |
| 28 | 1.3% |  | 0.7% |
| 29 | 8.0% |  | 5.1% |
| 30 | 5.6% |  | 6.5% |
| 31 | 0.9% |  | 1.4% |
| 32 | 1.4% |  | 11.1% |
| Round UVR Lineage Isolates | | | |
| 25R | 1.3% |  | 1.2% |
| 26R | 2.5% |  | 0.5% |
| 27R | 5.7% |  | 0.6% |
| 28R | 1.7% |  | 0.1% |
| 29R | 0.5% |  | 0.9% |
| 30R | 1.3% |  | 0.9% |
| 31R | 0.9% |  | 0.01% |
| 32R | 8.4% |  | 0.9% |
| Fuzzy UVR Lineage Isolates | | | |
| 25F | 8.1% |  | 0.5% |
| 26F | 1.1% |  | 0.1% |
| 27F | 7.0% |  | 0.4% |
| 28F | 8.7% |  | 0.04% |
| 29F | 2.7% |  | 0.5% |
| 30F | 1.2% |  | 0.2% |
| 31F | 2.3% |  | 0.1% |
| 32F | 0.4% |  | < 0.01% |
| Non-UVR Lineage Isolates | | | |
| 33 | 1.1% |  | 0.1% |
| 34 | 0.8% |  | 2.9% |
| 35 | 0.9% |  | 0.1% |
| 36 | 0.6% |  | 0.7% |
| 37 | 0.8% |  | 0.1% |
| 38 | 1.2% |  | 0.2% |
| 39 | 2.3% |  | 0.2% |
| 40 | 3.9% |  | 1.6% |

^a^ Percent survival of the *P. cichorii* 302959 ancestor was 0.7%.
